# A Simple Method Based on the Application of a CCD Camera as a Sensor to Detect Low Concentrations of Barium Sulfate in Suspension

**DOI:** 10.3390/s110100864

**Published:** 2011-01-13

**Authors:** Rodrigo Caciano de Sena, Matheus Soares, Maria Luiza Oliveira Pereira, Rogério Cruz Domingues da Silva, Francisca Ferreira do Rosário, Joao Francisco Cajaiba da Silva

**Affiliations:** 1 Universidade Federal do Rio de Janeiro (UFRJ), Instituto de Química, Avenida Athos da Silveira Ramos, 149 Bloco A–7^0^ andar, Rio de Janeiro, 21941-909, Brazil E-Mails: rcsena@inmetro.gov.br (R.C.S.); matheus@peq.coppe.ufrj.br (M.S.); luizadot@gmail.com (M.L.O.P.); cruz.domingues@yahoo.com.br (R.C.D.S.); 2 Instituto Nacional de Metrologia, Normalização e Qualidade Industrial, Xerém, Duque de Caxias, RJ, Brasil; 3 Centro de Pesquisas e Desenvolvimento Leopoldo Américo Miguez de Mello, Rio de Janeiro, RJ, Brasil; E-Mail: frosario@petrobras.com.br

**Keywords:** imaging, precipitation, CCD camera, turbidity

## Abstract

The development of a simple, rapid and low cost method based on video image analysis and aimed at the detection of low concentrations of precipitated barium sulfate is described. The proposed system is basically composed of a webcam with a CCD sensor and a conventional dichroic lamp. For this purpose, software for processing and analyzing the digital images based on the RGB (Red, Green and Blue) color system was developed. The proposed method had shown very good repeatability and linearity and also presented higher sensitivity than the standard turbidimetric method. The developed method is presented as a simple alternative for future applications in the study of precipitations of inorganic salts and also for detecting the crystallization of organic compounds.

## 1. Introduction

Scale deposition is a common and serious problem in the oil and gas industry. Scale is a set of deposits that can develop near the wellbore and is capable of reducing the oil production. Scaling can also cause deposits in down-hole pumps, tubing, casing flow-lines, tanks and other production-equipment and facilities. Scale formation leads to a decrease of the oil flow and sometimes even completely blocks its passage. These phenomena may cause production-equipment failure, increased maintenance cost and decrease in production efficiency [1–11].

Barium sulfate (BaSO_4_) and calcium carbonate (CaCO_3_) are the most common scale-producing chemicals. The deposits formed by barium sulfate are problematic because this salt is difficult to remove once formed and the costs associated with its removal are high. In the offshore oil and gas production, seawater is injected into the reservoir for pressure maintenance and to improve secondary recovery. Water that exists in the reservoir, called formation water, usually contains barium ions capable of reacting with sulfate anions present in the seawater, producing insoluble barium sulfate. [2,3,12].

Offshore production in deep seawater brings with it a number of additional challenges for controlling inorganic scale formation. The cost of intervention for scale removal in deep seawater is high and requires more accuracy for scale prediction and risk assessment. In order to evaluate the most practical and economical method to avoid or minimize barium sulfate precipitation (use of chemical scale inhibitors or performing seawater desalination), it is necessary to understand the barium sulfate precipitation process and identify the conditions that favor it. The kinetic studies of barium sulfate precipitation reported in literature are based on measuring solution properties, such as conductivity [8,13,14], offline quantitative analysis of ions or in line techniques such as turbidimetric measurement [11,15–18].

The determination of kinetics parameters of precipitation reactions is generally based on the classical theory of nucleation or population balance [1,19–22]. The detection of the beginning of nucleation is completely dependent on the sensitivity of the method employed to evaluate crystallizations [23]. Therefore, the chosen experimental technique plays an important role in the determination of the kinetics of precipitation. The response of sensors based on measurements of the solution properties, such as conductivity or ionic activity, which can be influenced by the solution composition (salinity, solvents) while measurements of turbidity using in-line sensors demands a minimum particle size and amount for detection.

This work reports the development of a method based on a video image analysis designed to achieve an optimized performance in the detection of low concentrations of barium sulfate in suspension. The method employs a low cost webcam as a sensor. The performance of the method has been evaluated by comparison with the turbidimetric method. The developed method is presented as a simple alternative for future applications in the study of precipitations of inorganic salts and crystallizations.

### 1.1. Sensors Based on Image Analysis

The use of Complementary Metal Oxide Semiconductor (CMOS) and Charge Coupled Device (CCD) cameras has been widely introduced in analytical chemistry for different reasons such as, fast image capturing, stable background and good linearity [24]. These sensors are capable of converting the intensity of light that focuses on it in digital storable values as bits. The analytical response generates an image representing the patterns of colors Red (R), Green (G) and Blue (B). These patterns are named as RGB 8 bits for each channel, totaling 256 levels. The combination of the three matrices (R, G and B) allows the acquisition of 16 million colors [25,26].

Different methodologies employing image analysis have been described in the literature. Maleki *et al.* [27] employed a digital camera as a sensor for simultaneous determination of Al(III) and Fe(III) in alloys using chrome azurol S (CAS) as chromogenic reagent. Gaiao *et al.* [25] proposed a novel instrumental detection technique for titration based on digital images, while Lyra *et al.* [25] proposed a similar method for measurement of lithium, calcium and sodium through the radiation emitted by the analyte into an air-butane flame. Hernández *et al.* [29] proposed a technique based on image analysis for a real-time assessment of coffee roasting processes. Shirshov *et al.* [30] described an approach employing a CCD camera as a sensor for recognizing volatile alcohols. Simon *et al.* [23] proposed a method based on external bulk video imaging. The method was proposed for metastable zone identification in food and pharmaceutical crystallization processes and had shown a good performance when compared to Focused Beam Reflectance Method (FBRM) and ultra-violet visible spectroscopy. Novales *et al.* [31] described a method for characterization of emulsions and suspensions by video image analysis while Simon *et al.* [32,33] proposed a methodology based on external bulk video image to monitoring and detection of nucleation. Additionally, a CCD camera has been used as a detector in clinical analysis and showed high detection sensitivity and good linear correlation [34,35].

The rapid improvements in digital camera technology provide the opportunity for the development of new methodologies employing digital cameras as an analytical sensor with high sensitivity, robustness, fast and low cost for implementation that reduces the analysis time.

## 2. Experimental Section

### 2.1. Synthesis of Barium Sulfate

Analytical grade BaCl_2_ and Na_2_SO_4_ solutions were used to synthesize BaSO_4_. Distilled water was used for preparing the solutions. The used procedure was as follows: in a 1 L Erlenmeyer flask containing 250 mL of barium chloride 0.15 M at 25 °C was added 250 mL of sodium sulfate 0.15 M solution slowly and under constant stirring. The formed suspension was stirred for two hours and then was kept at rest for 24 hours for decantation. The crystals formed were filtered off and dried at 40 °C for 72 hours. The crystals were sieved using a shaker-equipped sieve set (Viatest GMBH). The crystals retained on the 53 μm sieve were separated.

### 2.2. Preparation of Barium Sulfate Suspensions

Barium sulfate saturated solution was prepared by adding 0.10 g of BaSO_4_ in 1,000 mL of distilled water in a 2 L flask. The suspension was kept stirred for 2 hours at 25 °C and then filtered using a Millipore system with a 0.45 μm membrane. Suspensions with 2.5, 5, 7.5, 10, 15, 25, 100, 150, 200 and 250 mg/L of barium sulfate were prepared using the saturated solution. Measurements were carried out in a double walled AP01 reactor of 1 L capacity connected to a Mettler RC1e calorimeter. An overhead stirrer at 300 rpm was used to agitate the system. The stirring rate was chosen to be high enough to guarantee that particles were well suspended, but low enough to avoid bubbles formation. The experiments were carried out at 25 °C.

### 2.3. Experimental Setup

**Figure 1.**
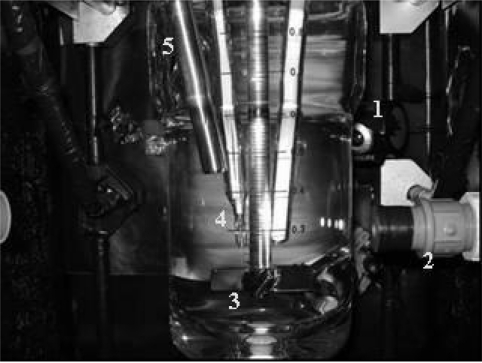
Set-up of the measuring system (1-webcam; 2-light source; 3-stirrer; 4-temperature sensor, 5-turbidity sensor).

The experimental set up is presented in Figure 1. It consists of a double walled glass reactor with 1 L capacity equipped with an lnPro8200 inline turbidity sensor (Mettler-Toledo) with a Trb8300 signal transmitter. The experiment temperature was controlled using a RC1 reactor calorimeter. A PC webcam (Philips SPC 900NC) was used to capture images in real time. In order to avoid interferences by external light and to maintain the CCD noise under controlled conditions, the reactor was enclosed in a black box during the measurements. A tungsten halogen light source (OSRAM—50 W, color temperature of 3,000 K) was used for the illumination. A system with optical-fiber (Olympus—ALS—150U) was utilized to guide the light from the source to the box.

### 2.4. Software for Data Acquisition and Evaluation

In order to perform video image analysis evaluation, a software named Masterview was developed. It is written in Delphi and was devoted to capture images in real time from a PC webcam by evaluating changes in the components’ RGB color pixel by pixel. The software allows the analysis of the whole image or the user can define a specific region previously selected from an image. The software automatically saves the coordinates of the delimited region for all digital images and calculates the R, G and B values averaging all pixels.

### 2.5. Turbimetric Measurements

The turbidimetric measurements and the video image analysis were carried out at the same time. The output current of the turbidity sensor was transferred to computer by using a data acquisition board (USB-6009 data acquisition device—National Instruments). Labview was used for data acquisition.

## 3. Results and Discussion

### 3.1. Method Optimization

Most commercially available web cameras have automatic adjustments depending on light conditions. This feature is not adequate for methods based on digital video image analysis because its principle is based on changes in color of the object or the analyzed medium. The possibility of changing the camera settings is advisable to guarantee the adequate quality of the results furnished by the RGB analysis method. In this study, a Philips SPC 900NC webcam was used which features this facility and also capable of obtaining images under low luminosity conditions (1 lux).

The webcam was configured to capture 24-bits digital images at a rate of 30 images/s and 640 × 480 pixels of spatial resolution. In order to optimize the analytical methodology the camera has been configured in the following way: gamma 40%, saturation 60%, shutter-speed = 60%, face tracking = off, digital zoom = off, picture enhancer = off, full automatic control = off, auto exposure = off, auto white balance = off, black & white = off, backlight compensation = off. It was observed that the main parameters capable of influencing the sensitivity of the method were brightness and gain. These parameters were optimized for assessing the sensitivity of the method for detecting small amounts of barium sulfate in suspension.

The analytical data that a digital camera returns are a standard trichromatic response (red, blue and green), where the output signal ranges from 0 to 255 for each channel [27]. When the output signal reaches the maximum value (255) the CCD sensor is unable to evaluate further changes in the color components.

**Figure 2.**
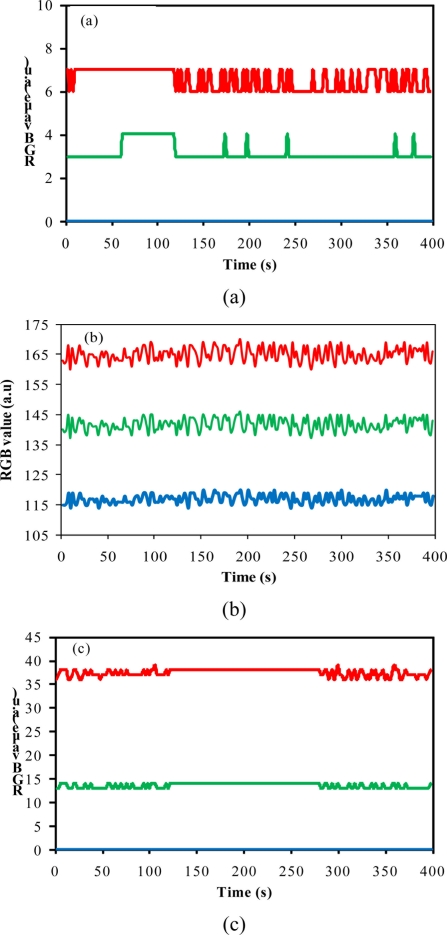
Effect of camera configurations on the output RGB signal obtained from a blank solution: **(a)** configuration A; **(b)** configuration B; **(c)** configuration C.

The comparison between the output RGB signals furnished by the software and the camera configuration is presented in Figure 2. A blank solution was used as reference. All experiments were carried out under the same luminosity conditions. Three configurations were considered for evaluation of the method performance: configuration A (brightness 60%; gain 50%), configuration B (brightness 100%; gain 100%) and configuration C (brightness 60%, gain 100%).

In Figure 2, it can be seen that when high levels of brightness and gain were used, an increase in the noise levels was observed [Figure 2(b)]. A high level of the output signal is not desirable because it reduces the range available for measurements considering that the available range for RGB is 0–255 units. An increase in the noise also reduces the method sensitivity. The direct inspection of the results presented in Figure 2 shows that the system presented good stability during the data acquisition, independently of the camera configuration employed. For configurations A and C, the output signal obtained from the blue channel was zero.

**Figure 3.**
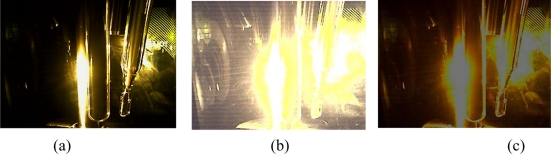
Images obtained from a blank solution by using different camera configurations: **(a)** camera configuration A; **(b)** camera configuration B; **(c)** camera configuration C.

The images obtained from the blank solution by using the camera configurations presented in Figure 2 are shown in Figure 3. The region delimited in Figure 3(a) was selected for data acquisition and was chosen in a way that the interference caused by the reflection of incident light on the sensors was minimized.

Observing the results presented in Figure 3, it is clear that the use of the camera configuration B is strongly affected by light reflections over the reactor inserts. This may explain the increased noise observed in the graph presented in Figure 2(b). Considering that the software allows the selection of a specific area of the whole image to perform the RGB analysis, the camera configurations A and C were used for evaluating the method performance for detecting low concentration of barium sulfate in suspension.

### 3.2. Relationship between RGB Level and Concentration of Barium Sulfate in Suspension

**Figure 4.**
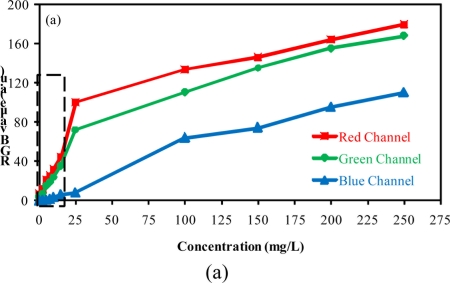

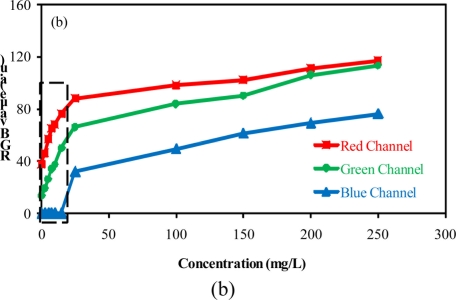
Red, Green and Blue channel levels for different concentrations of barium sulfate in suspension: **(a)** configuration A, **(b)** configuration C.

Figure 4 presents the relationship between the value of each color component and the concentration of barium sulfate in suspension using the camera configurations A and C. All measurements were carried out in triplicate.

The results presented in this figure show the effect of camera configurations on the linearity and the sensitivity of the method. They show that the blue component presented less sensitivity. Furthermore, it was observed an alteration in the relationship between the output RGB signal and the barium sulfate concentration in suspension when compared to the results obtained for low (≤15 mg/L) and high (≥15 mg/L) concentrations. Two curves with different slopes can be observed (dotted line in Figure 4 delimits the region where the slopes undergo changes).

**Figure 5.**
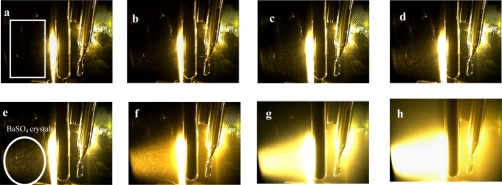
Images of barium sulfate suspensions of different concentration: **(a)** blank solution, **(b)** 2.5 mg/L, **(c)** 5 mg/L, **(d)** 7.5 mg/L, **(e)** 10 mg/L, **(f)** 25 mg/L, **(g)** 100 mg/L, **(h)** 250 mg/L.

Aiming at explaining the different slopes observed in the graphs, the images for different concentrations of suspended barium sulfate particles detected by using the camera configuration A, are presented in Figure 5. The data acquisition was carried out in the region delimited in Figure 5(a), where the interference caused by the reflection of incident light on the sensors was minimized.

The results presented in Figure 5 shows the color intensity variation in function of the solids concentration in suspension. Single and multiple light scattering are physical process that can occur when the radiation interact with the BaSO_4_ crystals in suspension. The acquired images for concentration from 2.5 mg/L to 15 mg/L [Figure 5(b–e)] shows that color intensity changes when compared to solutions with concentration above 15 mg/L [Figure 5(f–h)]. This behavior is apparently caused by the predominance of multiple light scattering in solutions with high concentrations of solids.

### 3.3. Limits of Detection and Quantification

**Figure 6.**
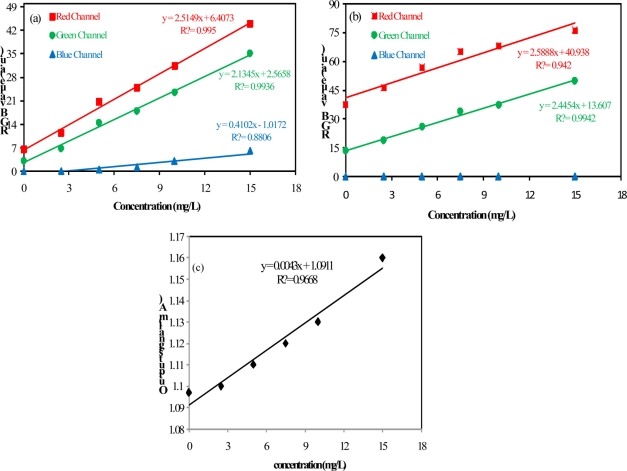
Analytical curves: **(a)** video image analysis—configuration A; **(b)** video image analysis—configuration B; **(c)** output of the turbidity sensor.

Regarding the data shown in Figure 4, it can be seen that the method sensitivity is bigger for measurements of low concentrations of barium sulfate in suspension (the slope for low concentration is higher). Based on these observations the limit of detection (LOD) and the limit of quantification (LOQ) were evaluated by concentrations below 15 mg/L. The analytical curves for concentrations below 15 mg/L are presented in Figure 6.

The analytical performance of the method was assessed by using the LOD and LOQ. These data presented in Table 1, were calculated by using the standard deviation of the blank solution measurements (s_b_), obtained from three independents solutions, and the slopes of the analytical curves (β), according to the following equations:

(1) $$\mathrm{LOD}=\frac{{3s}_{b}}{\beta }$$

(2) $$\mathrm{LOQ}=\frac{{10s}_{b}}{\beta }$$

As presented in Table 1, both configurations that were used in the video image analysis method have yielded similar results in relation to LOD and LOQ. The analytical curve obtained from the red channel data presented the largest slope, however, the noise associated with the green component was smaller and the combination between S_b_ and β indicated better results when using the green component. The proposed method presents better performance in terms of the figures of merit LOD and LOQ in relation to the turbidimetric method. Additionally, it is non-invasive and this feature avoids contamination issues and interferences in the measurements.

## 4. Conclusions

The results presented have demonstrated that video image analysis method is a useful tool for detecting low concentrations of barium sulfate in suspension. The methodology has low limits of detection and quantification and shown a better performance in comparison to the standard turbidimetric method. The results obtained demonstrate the analytical applicability of the proposed methodology. Compared to other measurements apparatus, the proposed method provides advantages especially in terms of its simplicity and low cost for implementation, in view of possible automation, this would allow reducing the study times and conducting various experiments at same time.

## Figures and Tables

**Table 1.** LOD and LOQ of the video image analysis method and turbidimetric method.

	Video Image Analysis Method				Turbidimetric Method
	Configuration* A		Configuration* C		
	R	G	R	G	
LOD (mg/L)	0.60	0.51	0.84	0.70	13.3
LOQ (mg/L)	1.99	1.70	2.80	2.34	44.3

* blue channel was not evaluated.
